# Non-Classical Inhibition of Carbonic Anhydrase

**DOI:** 10.3390/ijms17071150

**Published:** 2016-07-16

**Authors:** Carrie L. Lomelino, Claudiu T. Supuran, Robert McKenna

**Affiliations:** 1 Department of Biochemistry and Molecular Biology, University of Florida, Gainesville, FL 32611, USA; clomelino@ufl.edu; 2 Neurofarba Department, University of Florence, Piazza di San Marco, Firenze 50019, Italy; claudiu.supuran@unifi.it

**Keywords:** carbonic anhydrase, carboxylate, coumarin, non-classical

## Abstract

Specific isoforms from the carbonic anhydrase (CA) family of zinc metalloenzymes have been associated with a variety of diseases. Isoform-specific carbonic anhydrase inhibitors (CAIs) are therefore a major focus of attention for specific disease treatments. Classical CAIs, primarily sulfonamide-based compounds and their bioisosteres, are examined as antiglaucoma, antiepileptic, antiobesity, antineuropathic pain and anticancer compounds. However, many sulfonamide compounds inhibit all CA isoforms nonspecifically, diluting drug effectiveness and causing undesired side effects due to off-target inhibition. In addition, a small but significant percentage of the general population cannot be treated with sulfonamide-based compounds due to a sulfa allergy. Therefore, CAIs must be developed that are not only isoform specific, but also non-classical, i.e. not based on sulfonamides, sulfamates, or sulfamides. This review covers the classes of non-classical CAIs and the recent advances in the development of isoform-specific inhibitors based on phenols, polyamines, coumarins and their derivatives.

## 1. Introduction

Carbonic anhydrase (CA, EC 4.2.1.1) is a family of zinc metalloenzymes that catalyze the reversible interconversion of carbon dioxide and water to a bicarbonate and a proton. As such, CA activity is important for several physiological processes such as pH regulation, CO_2_ homeostasis, respiration, bone resorption, and tumorigenesis [1,2].

The CA family is subdivided into six classes based upon their structural fold, which also often correlate to their predominant organisms of expression. These classes include αCA expressed in vertebrates and algae; βCA in plants and prokaryotes; γCA in archaea; δCA and ζCA expressed in marine diatoms; and η in protozoa. In humans, there are 16 isoforms of αCA expressed that vary by localization and catalytic activity: CA I, CA II, CA III, CA VII, CA XIII are cytosolic; CA IV, CA IX, CA XII, CA XIV, CA XV membrane-bound; CA Va and CA Vb mitochondrial; and CAVI secreted in saliva and colostrum [3,4,5]. In addition, there are three catalytically inactive forms (CA VIII, CA X, and CA XI) referred to as CA-related proteins (CARPs) [5]. 

The αCA active site structure is conserved and is conically shaped with a zinc atom located at the base, which is coordinated by three histidine residues (His94, His96, His119) and a water/hydroxide ion. The catalytic mechanism of CA occurs in two steps. In the hydration direction, first there is a nucleophilic attack of carbon dioxide by the zinc-bound hydroxide ion, resulting in a zinc-bound bicarbonate that is subsequently displaced by a water molecule [4,6,7]. The enzyme active site is then regenerated to a zinc-bound hydroxide ion by a proton transfer mechanism from the zinc-bound water molecule to bulk solvent, facilitated by an ordered water wire within the active site and His64 acting as a proton donor/acceptor shuttle residue [8,9,10,11]. 

### 1.1. CA Inhibition

Various isoforms of CA have been identified as therapeutic targets for several diseases, see Table 1. Therefore, the design of isoform-specific inhibitors is studied for the development of new and improved treatments [6,12,13,14,15,16,17,18,19,20,21,22,23]. 

CA is classically inhibited by compounds with a sulfonamide-based (SO_2_NH_2_) zinc-binding group (ZBG) or their bioisosteres (sulfamates and sulfamides). Sulfonamides bind in a tetrahedral geometry, interacting directly with the catalytic zinc in their deprotonated form, and inhibit CA activity by displacing the zinc-bound water/hydroxide ion. There are currently several sulfonamide-based CA inhibitors clinically available for the treatment of glaucoma, edema, epilepsy, and altitude sickness [4]. 

**Figure 1:**
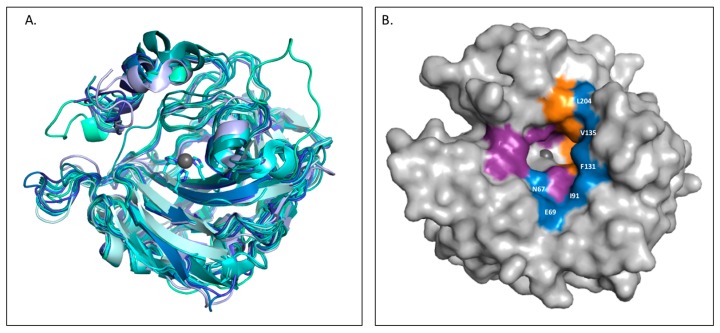
(**A**) Overlay of catalytically active human Carbonic anhydrase (CA) isoforms; (**B**) Surface representation of CA II with hydrophobic and hydrophilic regions colored orange and purple, respectively. Isoform-unique residues within the active site are colored blue and labeled.

However, the design of isoform-specific inhibitors is complicated by the structural homology between the 16 CA isoforms, which is particularly high within the active site (Figure 1A). Consequently, the available CA inhibitors act systemically and bind nonspecifically, causing a range of undesired side effects, such as fatigue and nausea, due to off-target inhibition. As CA II is the most physiologically abundant isoform, it is often regarded as the predominant off-target isozyme of which inhibition is to be avoided [3,4,24,25]. It has been suggested that nonspecific, classical carbonic anhydrase inhibitors (CAIs), such as acetazolamide, be delivered in large doses to increase the probability of inhibiting a disease-associated target isoform. However, the inhibitor distribution would be disproportionate in relation to the levels of isoform expression. As such, CA II would sequester the free inhibitor and prevent binding to the target isoform. Therefore, the development of isoform specific inhibitors will prevent side effects and improve the distribution of CA inhibitors. 

The design of isoform-selective compounds has been improved by the identification of a selective pocket in human CA active sites and further development of drug design approaches. The CA active site can be divided into two halves characterized by hydrophobic and hydrophilic residues. While active site residues surrounding the catalytic zinc are primarily conserved between isoforms, unique residues are located 10–15 Å from the zinc, designated as the selective pocket (Figure 1B) [26,27,28]. Therefore, compounds can be specifically designed to exploit interactions with these residues utilizing the tail method. In this approach, a lead compound with a high-affinity ZBG is identified and the “tail” of the drug modified in order to promote interactions with isoform-unique residues or alter the solubility and physico-chemical properties of the compound. Common variations include the length of the tail and class or characteristics of the chemical substituents used to derivatize the inhibitor [5,29]. Recently, the success of the tail method to produce isoform specific inhibitors was highlighted by the design of SLC-0111, a CA IX-specific inhibitor currently being tested in clinical trials for the treatment of breast cancer [30]. 

### 1.2. Non-Classical CA Inhibition

Although sulfonamide-based compounds have been studied as CAIs for several decades and several inhibitors are in clinical use, 3%–6% of the general population cannot be treated with such compounds due to sulfa allergies. Sulfonamide treatments can result in either a type 1 immunological reaction, commonly caused by immunoglobulin E (IgE) antibodies developed against the inhibitor, or a non-type 1 response in which metabolites of the inhibitor interact with a native protein or T cell to stimulate a response [31]. The incidence of adverse reactions to sulfonamide-based compounds increases with age and is more commonly exhibited in women [32]. Although reactions are most often seen to sulfonamide antibiotics, cases have been reported for allergic responses to non-antibiotic CAIs, such as acetazolamide. Side effects of commonly used sulfonamide CAIs cover a wide range from epidermis rash to nausea to anaphylactic shock or acute respiratory failure [33]. Therefore, there is a need for CAIs that are not only isoform-specific, but also non-classical so they are not based on a sulfonamide, sulfamate, or sulfamide ZBG. 

**Figure 2:**
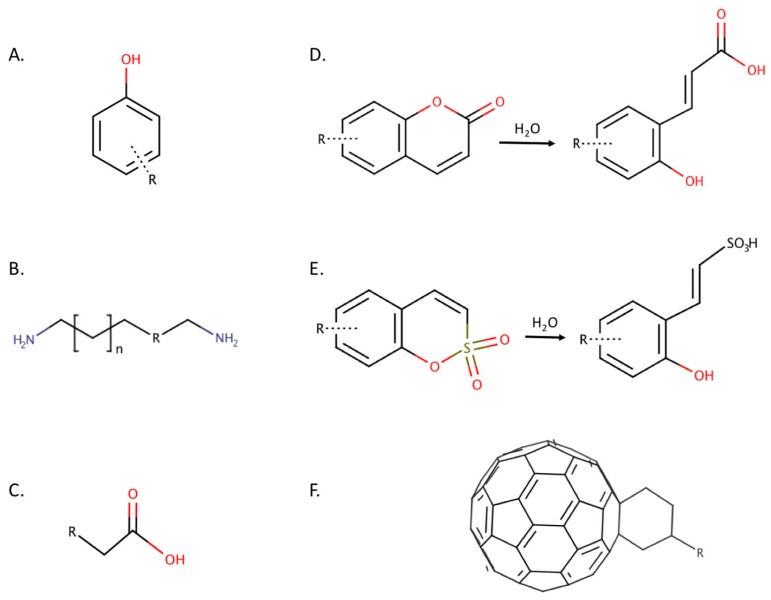
(**A**) Phenol; (**B**) Polyamine: *n* is an integer, R is either an N or C atom; (**C**) Carboxylic acid; (**D**) Coumarin and hydrolysis product; (**E**) Sulfocoumarin and hydrolysis product; (**F**) Fullerene derivative.

Several classes of compounds have recently been identified as non-classical CAIs, including phenols, polyamines, carboxylic acids, coumarins and their derivatives, in addition to fullerenes (Figure 2). While it is possible for non-classical inhibitors to maintain the traditional tetrahedral geometry by binding directly to the catalytic zinc, the classes covered in this review also participate in different binding mechanisms. For example, non-classical CAIs have been identified that inhibit CA catalytic activity by anchoring to the zinc-bound water/hydroxide ion or binding to the enzyme outside the active site, occluding the entry of substrate and preventing catalysis. 

## 2. Non-Classical Inhibitor Classes

### 2.1. Phenols

**Figure 3:**
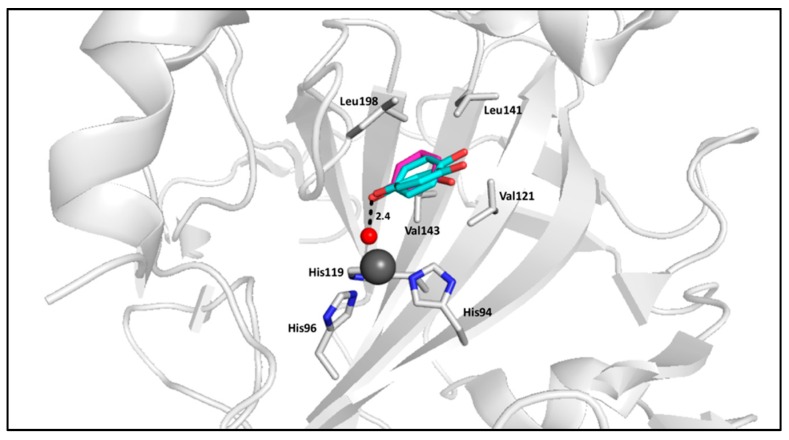
Phenol-based compounds benzene-1,4-diol (**cyan**) and resorcinol (**magenta**) in complex with CA II (**gray**) (PDBs: 4E3H and 4E49, respectively) [40].

Phenol is a CAI that acts as a competitive substrate with a micromolar affinity for CA in the forward hydration reaction [34]. The OH moiety anchors to the zinc-bound water/hydroxide ion through a hydrogen bond while the phenyl functional group participates in van der Waals interactions with the hydrophobic half of the active site, preventing the binding of carbon dioxide (Figure 3) [34,35]. Alternative to sulfonamide-based compounds, phenolic compounds have been shown to inhibit CA independent of the protonation state. Phenol inhibits all 13 catalytically active human isoforms with varying affinities, so phenol can be employed as a lead compound and new derivatives can be designed that improve isoform specificity [36]. As phenol itself is a rather compact structure, the inclusion of additional rings or other functional groups that lengthen the compound are expected to increase selectivity by promoting interactions with residues of the selective pocket. For example, the addition of a single chemical substituent, such as a carboxy moiety, has been shown to increase CA inhibition several fold with binding affinities in the low- to sub-micromolar range [37]. Phenolic ester inhibitors have been designed to increase the length of the compounds, exhibiting selective binding profiles of sub-micromolar affinities that are simultaneously poor inhibitors of off-target CA II [38].

Polyphenols and more structurally complex natural products are similarly shown to exhibit improved binding affinities in comparison to the lead compound, phenol [37]. Phenol-based natural product discovery provides a promising direction for CA inhibition since these compounds are derived from plants and are already ingested in the human diet, providing both a nontoxic and sulfur-free compound. Several of these phenol-based natural products show specificity for CA VII and are recognized to also play a role in antioxidant activity, which in combination with CA inhibitory activity could develop into novel treatments for neurodegenerative diseases [39].

### 2.2. Polyamines

**Figure 4:**
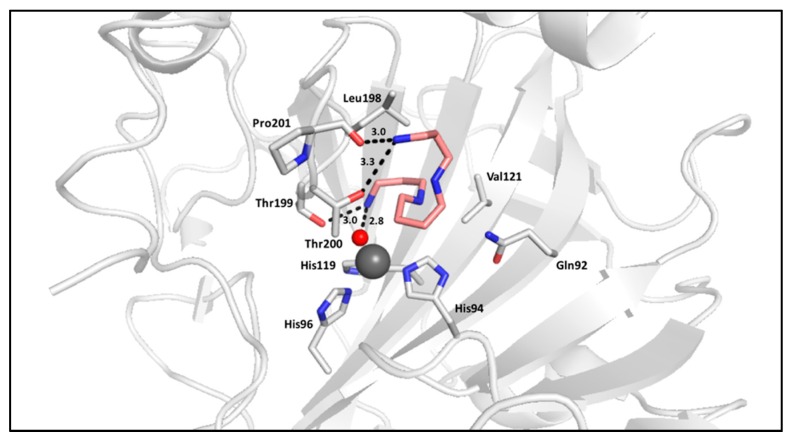
Spermine (**salmon**) in complex with CA II (**gray**) (PDB:3KWA) [41].

Polyamines are polycationic, aliphatic molecules that were originally expected to act as activators of CA due to the activating properties observed for amino acids and amines. However, activity assays determined several polyamines, such as spermine, spermidine, and their derivatives, exhibit inhibitory properties of CA. The crystal structure of spermine in complex with CA II elucidated the binding mechanism for polyamines; similar to that of phenolic compounds, a terminal ammonium group anchors to the zinc-bound water/hydroxide ion through a hydrogen bond. Polyamine binding is also unique because the compound binding relies on a network of hydrogen bonding, with key interactions occurring between the inhibitor and Thr199, a residue conserved between all isoforms, and the other terminal amine with active site residues. Polyamines, such as spermine in adduct with hCA II, are observed to coil within the active site with the aliphatic portion stabilized by van der Waals interactions (Figure 4) [41]. 

Variations in the length of the aliphatic chain and the number of amine substituents are seen to affect CA binding affinities between the different isoforms, indicating the potential of polyamine compounds to be developed into selective CAIs. In addition, the polycationic nature of polyamines makes them unable to pass cell membranes, so these inhibitors can be used to target CA isoforms with extracellular catalytic domains. Therefore, polyamines show therapeutic potential in relation to the inhibition of cancer-associated CA IX and CA XII, which is highlighted by the selectivity of natural product polyamine fragments over cytosolic off-target isoforms [42]. 

### 2.3. Carboxylic Acids

**Figure 5:**
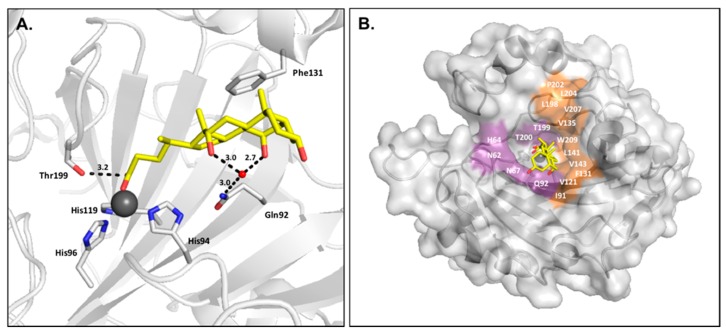
(**A**) CA II (**gray**) in complex with cholic acid (**yellow**) (PDB:4PXX); (**B**) Surface representation of CA II in complex with cholic acid (**yellow**).

Carboxylic acids represent a class of compounds that inhibit metalloenzymes through various mechanisms of action, such as coordinating to the metal ion in a mono- or bidentate manner. This is also the case for CAs, for which a number of inhibition mechanisms have been observed [43]. First, carboxylate compounds can directly bind to the catalytic zinc and displace the bound water/hydroxide ion, as seen in classical sulfonamide inhibition (Figure 5). 

**Figure 6:**
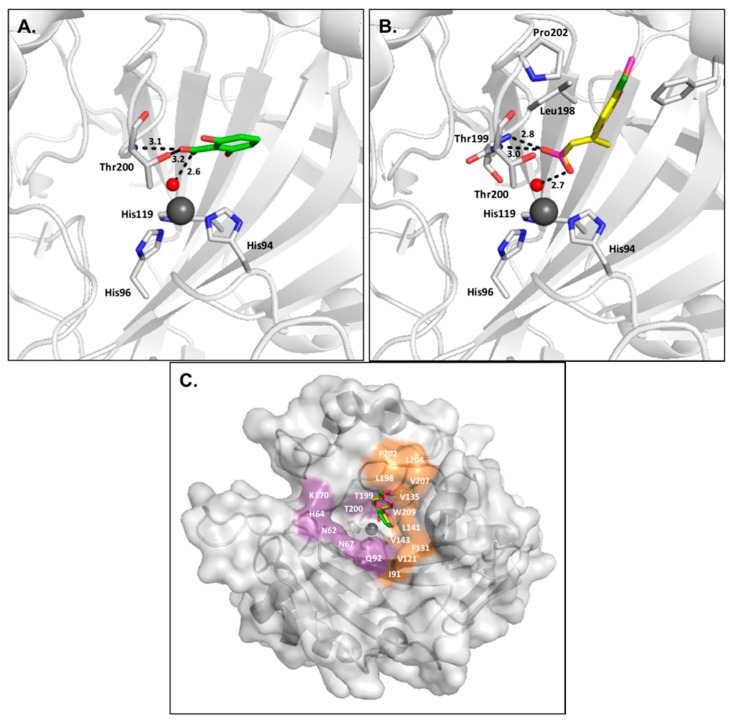
(**A**) CA II (**gray**) in complex with 2,6-dihydroxybenzoic (**green**, PDB:4E3F) [40]; (**B**) CA II (**gray**) in complex with ligands (*E*)-3-(4-methoxyphenyl)but-2-enoic acid (**magenta**) and (*E*)-3-(4-chlorophenyl)but-2-enoic acid (**yellow**) (PDBs: 5FNM and 5FLS, respectively) [44]; (**C**) Overlay of carboxylic acid–based compounds in CA II surface.

Secondly, some carboxylates anchor to the zinc-bound water/hydroxide ion through a hydrogen bond, similar to the binding mechanism seen in phenol-based compounds (Figure 6). However, these inhibitors are predicted to bind primarily in their anionic state [40]. 

**Figure 7:**
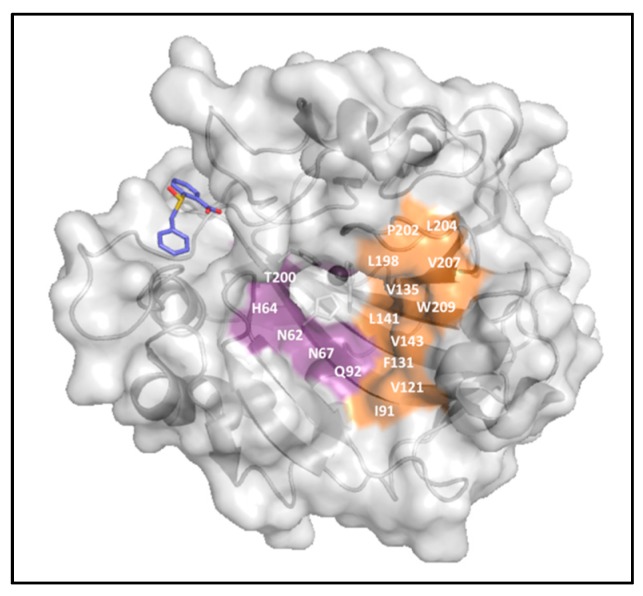
The 2-[(*S*)-benzylsulfinyl] benzoic acid (**periwinkle**) in complex with CA II (**gray**) (PDB:4QY3) [45].

Lastly, one carboxylate derivative was found to bind CA outside the active site, in a pocket adjacent to the entrance (Figure 7). This binding blocks His64, the proton shuttle residue, in its “out” conformation, leading to the inhibition of catalytic activity [45]. 

The scaffold of carboxylic acid–based inhibitors can vary in both size and chemical properties, allowing interactions with either the hydrophobic or hydrophilic half of the active site, in addition to isoform-unique residues of the selective pocket. Furthermore, the orientation of functional groups in relation to the carboxylic acid ZBG has been shown to be an important factor in binding due to the possibility of steric hindrance, further promoting selectivity based upon the size of amino acids lining the active site cavity [46]. Compounds that extend further from the active site have been shown to increase the binding affinity of carboxylic acid–based inhibitors over 100 fold, highlighting the importance of interactions with the selective pocket residues. For example, structures of CA II in complex with butenoic acid inhibitors (PDB:5FNM and 5FLS) exhibit Ki values between 700–900 µM, while a more compact benzoic acid derivative (PDB:4E3F) only has a Ki of approximately 5 mM. As these three compounds all anchor through the zinc-bound water/hydroxide ion and form hydrogen bonds with conserved residue Thr199, the additional van der Waals interactions formed by the butenoic acid derivatives with side chains in the hydrophobic half of the active site dictate this increase in specificity (Figure 6).

Several other scaffolds of carboxylic acid compounds have been discovered that exhibit isoform specificity. For example, inhibitors containing a cyclic imide scaffold selectively inhibit the cancer-associated isoforms CA IX and CA XII over off-target, cytosolic CA I and CA II. These carboxylic acid compounds exhibit more selective inhibition for the transmembrane CA isoforms than sulfonamide inhibitors containing the same cyclic imide scaffold [47]. Inhibitors with heteroaryl-pyrazole and hydroxy- oxoindolin-ylidene scaffolds show selectivity for CA I over CA II. CA I is studied as a target for the treatment of diabetic macular edema, a condition which left untreated can lead to loss of eyesight [46]. Lastly, carboxylic acid compounds incorporating a phthalic anhydride/phthalimide scaffold show selectivity for CA VII over CA I and CA II. As CA VII activity has been associated with epileptic seizures, CA VII selective inhibitors present a new method of treatment [48]. 

### 2.4. Coumarins

**Figure 8:**
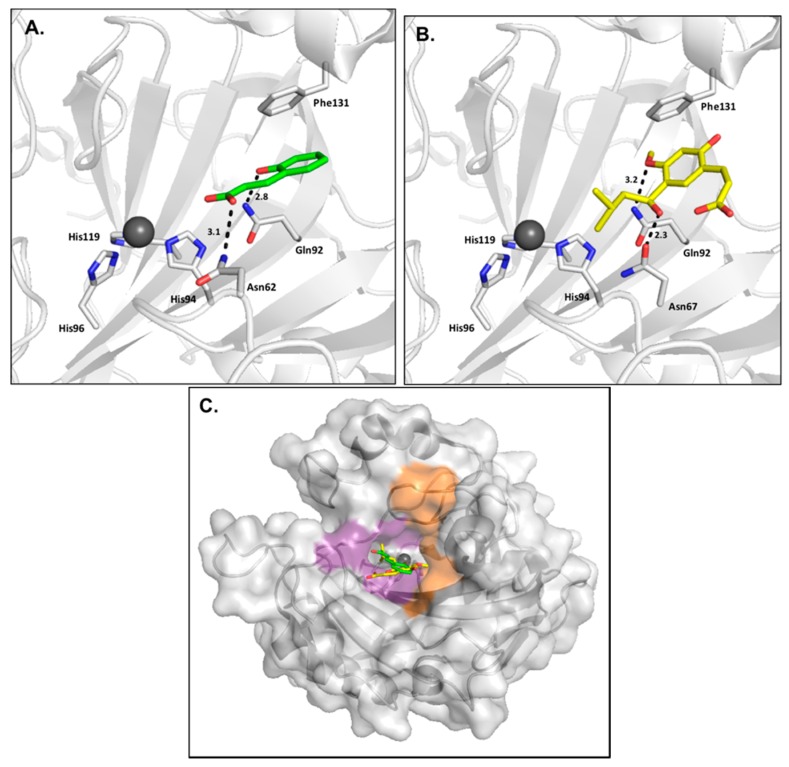
(**A**) Trans-2-hydroxycinnamic acid (**green**) in complex with CA II (**gray**) (PDB:5BNL) [50]; (**B**) (2*Z*)-3-{2-hydroxy-5-[(1*S*)-1-hydroxy-3-methylbutyl]-4-methoxyphenyl}prop-2-enoic acid (**yellow**) in complex with CA II (**gray**) (PDB:3F8E) [49]; (**C**) Overlay of coumarin inhibitor hydrolysis products in CA II surface.

Coumarins were first discovered through a general screen of natural product CAIs and quickly became of interest due to the observance of CA inhibition with micromolar to nanomolar affinity for all active αCA isoforms despite the lack of a canonical ZBG. Coumarins are unique from many other CAIs because they are considered prodrugs that can only bind the enzyme in the form of their hydrolysis product [49,50]. Coumarins are proposed to first bind in the active site, undergo hydrolysis due to the esterase activity of CA, and then reorient to prevent steric hindrance. Due to this required chemical transformation, coumarins are considered suicide compounds and their inhibitory properties are time-dependent [50]. The hydrolyzed form of the inhibitor binds at the entrance of the active site, blocking substrate entry and preventing catalysis (Figure 8) [49,50]. Since coumarins bind near or within the selective pocket where the majority of unique residues are located, this class provides a strong scaffold for the design of isoform selective inhibitors. It has been demonstrated that isoform specificity improves with the addition of chemical substituents to the coumarin scaffold, with the significance of improvement dependent on the number and chemical nature of the functional groups [49]. 

Coumarin-based CAIs are commonly studied in relation to the tumor-associated isoforms, CA IX and CA XII. For example, the design of 7-substituted coumarins with aryl-triazole substituents utilizing click chemistry led to compounds that exhibit selective inhibition in vitro in relation to cytosolic CA I and CA II [51]. Furthermore, the development of 7-glycosyl coumarin inhibitors that show not only selective inhibition profiles in vitro, but also exhibit therapeutic effects in mouse models of metastatic breast cancer highlight the strong potential for the use of coumarin CAIs in combination with standard chemotherapies as a novel cancer treatment [52]. 

### 2.5. Sulfocoumarins

**Figure 9:**
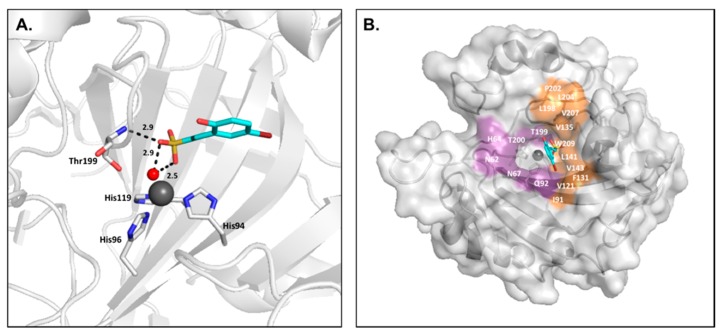
(**A**) CA II (**gray**) in complex with sulfocoumarin inhibitor (*E*)-2-(5-bromo-2-hydroxyphenyl) ethenesulfonic acid (**cyan**) (PDB:4BCW); (**B**) Surface representation of CA II in complex with sulfocoumarin inhibitor [53].

While studying coumarins as lead compounds, an isostructural class, sulfocoumarins, was discovered to significantly inhibit CA activity; several of these had a higher binding affinity to the cancer-associated CA IX in comparison to the above coumarins [53]. As seen in previous coumarin-based inhibitors, the sulfocoumarin compounds are hydrolyzed by the esterase activity of CA prior to binding. However, instead of binding outside the active site and occluding the entrance, the hydrolyzed product anchors to the zinc-bound water molecule/hydroxide ion through the sulfonic acid moiety (Figure 9) [53,54]. 

The addition of chemical substituents on the heterocyclic ring, and more importantly the position of these functional groups, has been shown to greatly affect the binding affinities to target CA isoforms. For example, the derivatization of the sulfocoumarin scaffold with a hydroxyl, methane-sulfonyl or benzyloxy moiety in position 6 of the 1,2-benzoxathiin 2,2-dioxide ring resulted in inhibitors significantly more specific to CA IX, exhibiting sub-micromolar binding affinities, than off-target CA I or CA II [53]. However, sulfocoumarins substituted in position 7 of this ring displayed specificity for CA II with low nanomolar binding affinities, which is better than acetazolamide. These 7-substituted sulfocoumarins therefore exhibit strong potential as a novel glaucoma therapy [54]. 

### 2.6. 2-Thioxocoumarins

**Figure 10:**
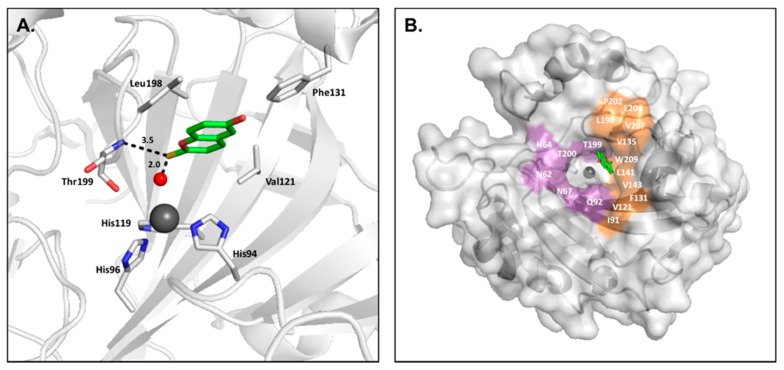
(**A**) CA II (**gray**) in complex with 6-hydroxy-2H-chromene-2-thione (**green**) (PDB:4WL4); (**B**) Surface representation of CA II in complex with thioxocoumarin inhibitor [55].

Though structurally related to the coumarin class, a 2-thioxocoumarin derivative was recently observed to bind in a region of the CA II active site distinct from the aforementioned coumarin compounds. In complex with CA II, the inhibitor was observed to anchor to the zinc-coordinated water molecule/hydroxide ion through the sulfur atom of the C = S functionality. Additionally, the compound bound was observed in its original state, not the hydrolysis product as seen for the previous coumarin inhibitors (Figure 10). The addition of the OH moiety on the 2-thioxocoumarin scaffold increases the affinity of the inhibitor for the membrane-bound, cancer-associated isoforms CA IX and CA XII several fold over off-target CA II [55]. 

### 2.7. Fullerenes

Fullerenes are C_60_ nanoscale carbon materials that can be derivatized to achieve selectivity for target proteins, such as CA. The diameter of a fullerene scaffold is similar to that of the CA active site, approximately 1 nm, so these compounds are predicted to inhibit CA by occluding the entrance of substrate into the active site. However, current structural studies with fullerene inhibitors are based on modeling and the exact binding location is not yet known. The addition of polar substituents to the fullerene scaffold has been shown to improve compound solubility. Additionally, the incorporation of amino acid or amine functional groups (typically common in CA activators) increases the number of interactions with CA active site residues, promoting inhibition with the bulky scaffold [56]. As fullerene compounds are expected to bind to regions outside the active site, similar to that of coumarins, these inhibitors also show strong potential for isoform-selective design.

## 3. Conclusions

Over the last few years, there has been a significant increase in focus on the development of non-classical CAIs for the treatment of multiple diseases due to the prevalence of sulfonamide allergies among the general population. The classes of non-classical inhibitors that show strong potential as lead compounds for isoform-specific drug design include phenols, polyamines, carboxylic acids, and coumarins and their derivatives. As these compounds can anchor to the zinc-bound water/hydroxide ion or bind outside the active site to block substrate entry, these classes exhibit binding mechanisms atypical from the classical sulfonamide CAIs.

## Figures and Tables

**Table 1:** Carbonic anhydrase (CA) related diseases with associated isoform targets.

Disease	CA Isoform Target
Glaucoma	CA II, CA IV, CA XII
Cancer	CA IX, CA XII
Epilepsy	CA VII
Antineuropathic pain	CA VII
Obesity	CA VA
